# A Study on the Effect of Surface Lysine to Arginine Mutagenesis on Protein Stability and Structure Using Green Fluorescent Protein

**DOI:** 10.1371/journal.pone.0040410

**Published:** 2012-07-09

**Authors:** Sriram Sokalingam, Govindan Raghunathan, Nagasundarapandian Soundrarajan, Sun-Gu Lee

**Affiliations:** Department of Chemical Engineering, Pusan National University, Busan, South Korea; Russian Academy of Sciences, Institute for Biological Instrumentation, Russian Federation

## Abstract

Two positively charged basic amino acids, arginine and lysine, are mostly exposed to protein surface, and play important roles in protein stability by forming electrostatic interactions. In particular, the guanidinium group of arginine allows interactions in three possible directions, which enables arginine to form a larger number of electrostatic interactions compared to lysine. The higher pKa of the basic residue in arginine may also generate more stable ionic interactions than lysine. This paper reports an investigation whether the advantageous properties of arginine over lysine can be utilized to enhance protein stability. A variant of green fluorescent protein (GFP) was created by mutating the maximum possible number of lysine residues on the surface to arginines while retaining the activity. When the stability of the variant was examined under a range of denaturing conditions, the variant was relatively more stable compared to control GFP in the presence of chemical denaturants such as urea, alkaline pH and ionic detergents, but the thermal stability of the protein was not changed. The modeled structure of the variant indicated putative new salt bridges and hydrogen bond interactions that help improve the rigidity of the protein against different chemical denaturants. Structural analyses of the electrostatic interactions also confirmed that the geometric properties of the guanidinium group in arginine had such effects. On the other hand, the altered electrostatic interactions induced by the mutagenesis of surface lysines to arginines adversely affected protein folding, which decreased the productivity of the functional form of the variant. These results suggest that the surface lysine mutagenesis to arginines can be considered one of the parameters in protein stability engineering.

## Introduction

Protein stability against non-physiological conditions, such as high temperatures, acidic or basic pH and detergents, is an important concern in industrial processes and in academic perspectives. Enhancing the protein stability has become increasingly critical due to the growing demand for using proteins as parts of bio-devices processed and operated under harsh conditions in addition to traditional enzymatic processes [1]–[3]. A deep understanding of the protein folding mechanism and protein structures has provided insights on the protein stability, which allowed the development of a number of protein engineering approaches to improve the protein stability [4]–[8]. For example, the protein stability can be enhanced by introducing more intra-disulfide bonds, designing more hydrophobic cores, or modifying the surface charges [9]–[12]. Nevertheless, further parameters related to protein stability need to be identified because the protein stability is a cooperative phenomenon of various interactions in protein.

Arginine and lysine are positively charged basic amino acids under physiological conditions and mostly exposed to the protein surfaces [13], [14]. The two amino acids on the protein surfaces play important roles in protein stability by forming ionic interactions and hydrogen bonds in the proteins as well as by interacting with water molecules [15], [16]. Although they both function as basic residues, the arginine residue provides the protein structure with more stability than lysine owing to its geometric structure. The guanidinium group in arginine allows interactions in three possible directions through its three asymmetrical nitrogen atoms (N^ε^, N^η1^, N^η2^), whereas only one direction of interaction is allowed by the basic functional group of lysine [17], [18]. This enables arginine to form a large number of electrostatic interactions, such as salt-bridges and hydrogen bonds compared to lysine, which presumably results in stronger interactions than the interactions generated by lysine. These effects have been demonstrated indirectly by comparing the salt-bridge numbers generated by arginine and lysine in a number of proteins as well as by analyzing the arginine to lysine ratio and salt-bridges in thermophilic and mesophilic proteins [19]–[23]. In addition to the geometric effect, the ionic interactions formed by arginine can be more stable than those of lysines, particularly under alkaline pH, due to the higher pKa of the basic residue in arginine than lysine [24].

The superior biochemical properties of arginine over lysine for the electrostatic interactions described above raises the following question. What will happen in terms of the protein stability and structure if lysine residues on a protein surface are changed to arginine? Specifically, this study focuses on the following: (1) whether the replacement of arginines with lysines on protein surface can induce more electrostatic interactions in the protein; (2) whether such mutagenesis can stabilize the protein; and (3) what type of stability can be improved by mutagenesis. By answering these questions, it might be possible not only to identify the effect of arginine and lysine on the protein stability and structure more directly but also to determine if such mutagenesis can be considered a way for protein stabilization.

**Figure 1:**
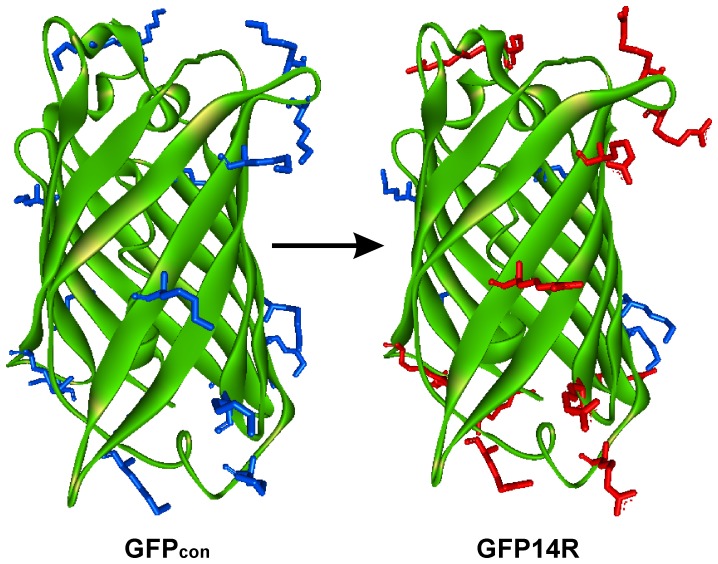
Structural representation of the GFP variants. Three dimensional cartoon representation of the GFP_con_ and GFP14R showing the surface lysines (blue) and the surface arginines (red) as sticks which were mutated in the GFP14R. Graphical image was generated using Discovery Studio Visualizer from Accelrys Software Inc.

This study attempted to answer above questions by mutating the lysine residues on a protein surface to arginine and examining its effect on the protein stability and electrostatic interactions. Green fluorescent protein (GFP), a protein with a fluorescent active site, where folding and unfolding can be monitored by its spectral properties [25], was used as the model system. GFP has been used mainly as a reporter system [26], but it is also employed in bio-devices, such as biochips and biosensors [27], [28], which require the stability of proteins under harsh conditions. In the first step of this study, the surface lysine residues of GFP were maximally mutated to arginines considering the foldability of the protein (Figure 1). In the second step, the stability of the variant was examined under a range of protein denaturation conditions, such as high temperatures, urea, alkaline pH and detergents. The effect of mutagenesis on the electrostatic interactions was also examined by analyzing the modeled structure of the variant. Finally, the possibility that mutagenesis of the surface lysine to arginine can be an approach to protein stability engineering is discussed.

## Results

### Analysis of the Electrostatic Interactions in GFP

The electrostatic interactions in GFP were analyzed using the wild type GFP crystal structure with PDB Id 1GFL [29]. Table 1 lists the salt-bridges and hydrogen bonds associated with the lysine and arginine residues in GFP. GFP has 20 lysine and 6 arginine amino acids corresponding to 8.4% and 2.5% of its total amino acid composition, respectively. For the 20 lysines, one, 8 and 11 are located in the helix, beta-strand and loop regions, respectively. For the 6 arginines, 4 and 2 are located in the beta-strand and loop regions, respectively. More than 25% of the side chains are exposed to the solvent for the 19 lysine and 5 arginine residues, which were considered to be exposed residues in this study. Lysine and arginine residues contribute to a total of 9 and 4 salt-bridge interactions, respectively. Residues, such as K45, K113, R109 and R122, form complex salt-bridges by interacting with more than one oppositely charged residue, whereas the remaining are simple salt-bridges by forming an ionic interaction with a single amino acid. The average number of salt-bridges per lysine and arginine were estimated to be approximately 0.45 and 0.66, respectively. An analysis of hydrogen bond interactions revealed the mean hydrogen bond interactions per lysine and arginine to be approximately 2 and 3, respectively. These results suggest that like most other proteins, the majority of lysine and arginine residues are exposed to the solvent, and arginine in GFP forms more electrostatic interactions than lysines.

**Table 1:** Salt-bridge and hydrogen bond interactions associated with the lysine and arginine residues in GFP.

Lysine	Solvent accessibility	Secondary structure	Salt-bridge interactions	Hydrogen bond interactions
K3	Exposed	Loop		
K26	Exposed	β-strand 2	D21	D21
K41	Exposed	β-strand 3		D36
K45	Exposed	β-strand 3	E213, D210	E32, E213
K52	Exposed	Loop		
K79	Exposed	Loop		Y74, H81
K85	Buried	Helix	D82	C70, S72, D82
K101	Exposed	Loop	D102	D102, Q177, L178
K107	Exposed	β-strand 5		N105, K126
K113	Exposed	β-strand 5	E111, E115	E115, V120
K126	Exposed	β-strand 6	D21	D21, G127
K131	Exposed	Loop		D102, D103, D133, D134
K140	Exposed	Loop		N135, E172
K156	Exposed	Loop		N159
K158	Exposed	Loop	D155	D155
K162	Exposed	β-strand 8		M153
K166	Exposed	β-strand 8		
K209	Exposed	Loop		H217
K214	Exposed	Loop		E213
K238*	Exposed	Loop	*	*
**Arginine**				
R73	Exposed	Loop		T38, V224, A226
R96	Buried	β-strand 4		T62, Y66, T108, Q183
R109	Exposed	β-strand 5	E111	E111, E124
R122	Exposed	β-strand 6	E115, E17	E17, E111, E115
R168	Exposed	β-strand 8		N146, H148
R215	Exposed	Loop	E213	E213, H217

* - three dimensional structure information is not known.

### Mutagenesis of Surface Lysines to Arginines

The GFP_mut3.1b_, a rapid folding GFP with mutations of S65G and S72A [30], was used as the target GFP protein and designated GFP_con_ in this report. In the initial mutagenesis study, all 19 surface-exposed lysine amino acids of GFP_con_ were mutated to arginines, which formed the variant designated GFP19R. When the variant was expressed in *Escherichia coli* at 37°C, the protein was completely misfolded *in vivo* (Figure S1). The expression of GFP19R at lower temperatures was performed to improve the folding efficiency of the variant but this approach was not effective in producing a soluble and folded protein (Figure S1).

**Figure 2:**
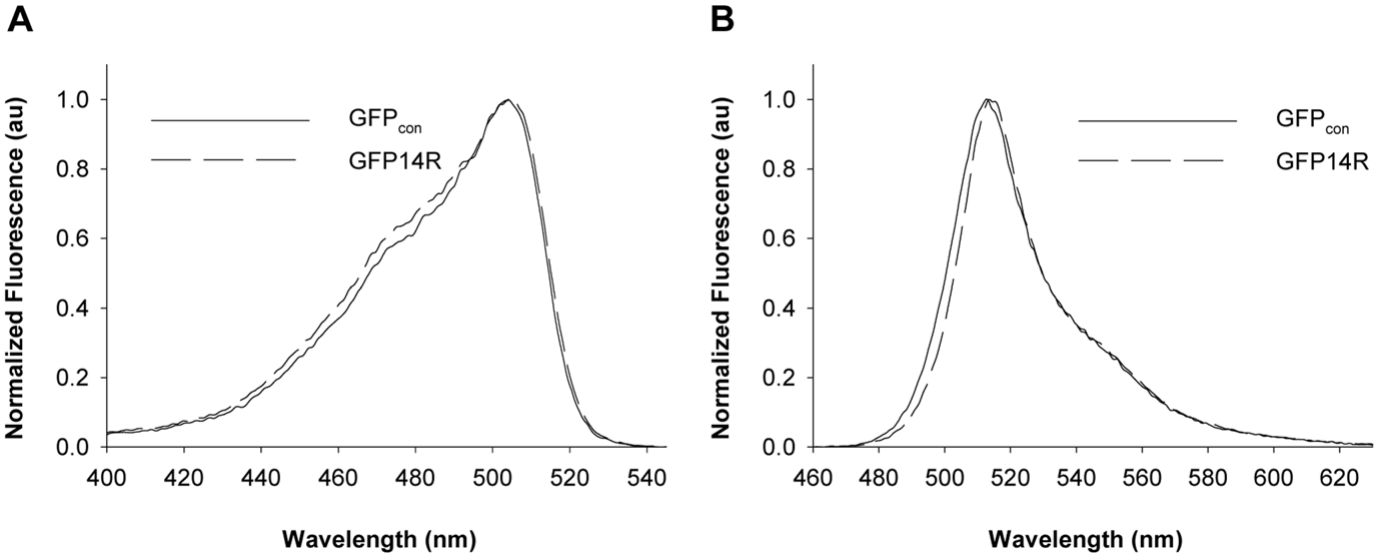
Spectral properties of the GFP variants. A) Excitation and B) Emission spectrum of the GFPcon and GFP14R. All the amplitudes were arbitrarily normalized to a maximum value of 1.0. (au – arbitrary units).

These results suggest that some of the lysine residues in GFP are essential for the proper folding of GFP. Previous report suggested the importance of the formation of a superstable core with the β-strands 1, 3, 4, 5 & 6 in GFP folding [31], which was used as a basis to overcome the misfolding problem of GFP19R. Because five lysines (K41, K45, K107, K113, K126) in GFP were involved in the β-strands of superstable core formation, the corresponding five arginine residues in GFP19R were back-mutated to lysines, which formed a variant GFP14R. The GFP14R were also mostly misfolded when expressed at 37°C (Figure S1), but soluble expression and whole cell fluorescence was detected by producing it at lower temperatures, 25°C (Figure S2) despite its much lower expression level than GFP_con_. This suggests that restoring the five lysines involved in the superstable core formation of GFP is effective in enhancing slightly the folding efficiency of GFP19R. A sufficient amount of GFP14R could be achieved by expressing it for longer periods at 25°C and the protein was purified for further studies (Figure S2). The purified GFP14R showed similar excitation and emission spectra to GFP_con_ (Figure 2), and its specific fluorescence intensity was also similar to that of GFP_con_ (Figure S3). These results suggest that the 14 Arg mutations introduced on the surface Lys residues had a substantial adverse effect on the folding efficiency of GFP, but did not affect the final GFP structure for its fluorescence activity significantly. Although the expression level of the variant GFP14R was much lower than that of GFP_con_, the ability of the variant to fold to its active form motivated us to carry out further studies on its stability.

### Effect of the Mutagenesis of the Surface Lysines to Arginines on GFP Stability

The GFP14R obtained from the above study contained 19 surface arginines and 5 lysines, whereas the GFP_con_ contained 19 surface lysines and 5 arginines. This suggests there is a prominent difference in the surface Arg to Lys ratio between the two proteins. To examine the effect of such a difference on the stability of GFP, the stability of the purified GFP14R and GFP_con_ were examined under four different protein denaturation conditions, i.e. high temperatures, urea addition, alkaline pH and detergent addition.

**Figure 3:**
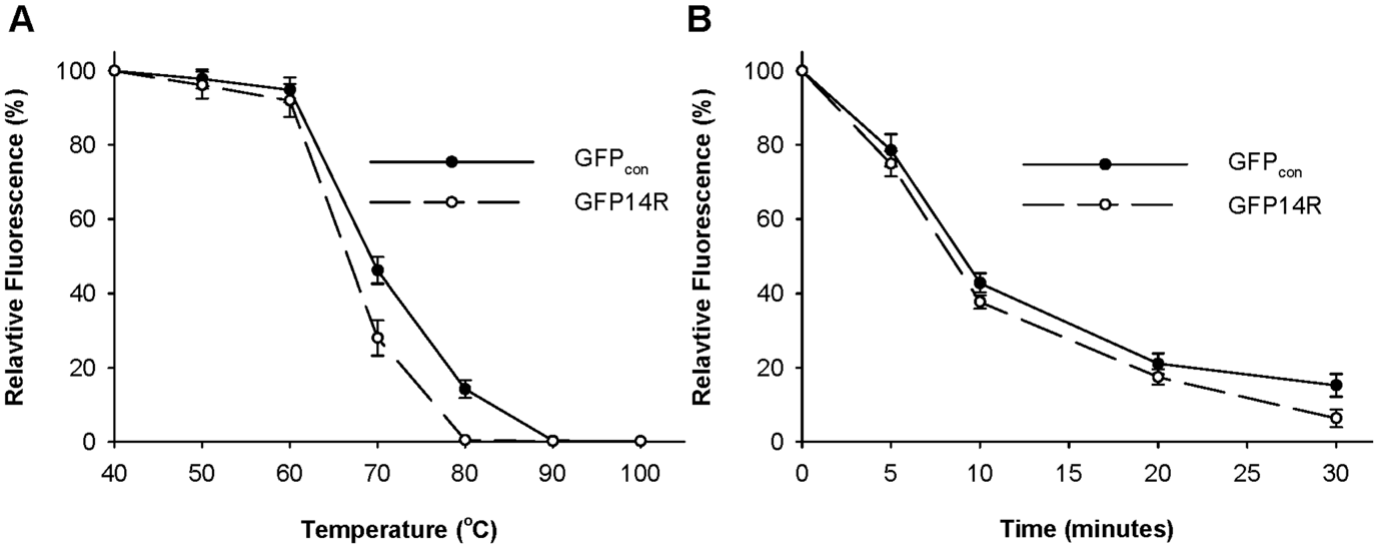
Effect of temperature on the stability of the GFP variants. A) Protein samples were incubated at different temperatures for 30 minutes and the remaining fluorescence was measured. The fluorescence at time zero at the respective temperatures was taken into 100%. B) Protein samples were incubated at 70°C for different time intervals and the remaining fluorescence was measured. The fluorescence at time zero was taken into 100%. (Error bar – Standard deviation of the three independent experiments).

First, the effect of temperature on the stability of the GFP proteins was investigated. A temperature-dependent assay was performed by incubating the GFP14R and GFP_con_ at a range of temperatures for 30 minutes, and measuring their fluorescence. As shown in Figure 3A, the GFP14R and GFP_con_ showed similar stability against temperature. They all tended to lose their fluorescence gradually after 60°C and lost their activity completely at 80°C. Both the GFP14R and GFP_con_ showed similar patterns when a time-dependent assay was performed at 70°C (Figure 3B). Their half-lives at 70°C were all estimated to be approximately 8 minutes. These results suggest that the introduced Arg mutations into the Lys residues did not affect the thermal stability of GFP.

**Figure 4:**
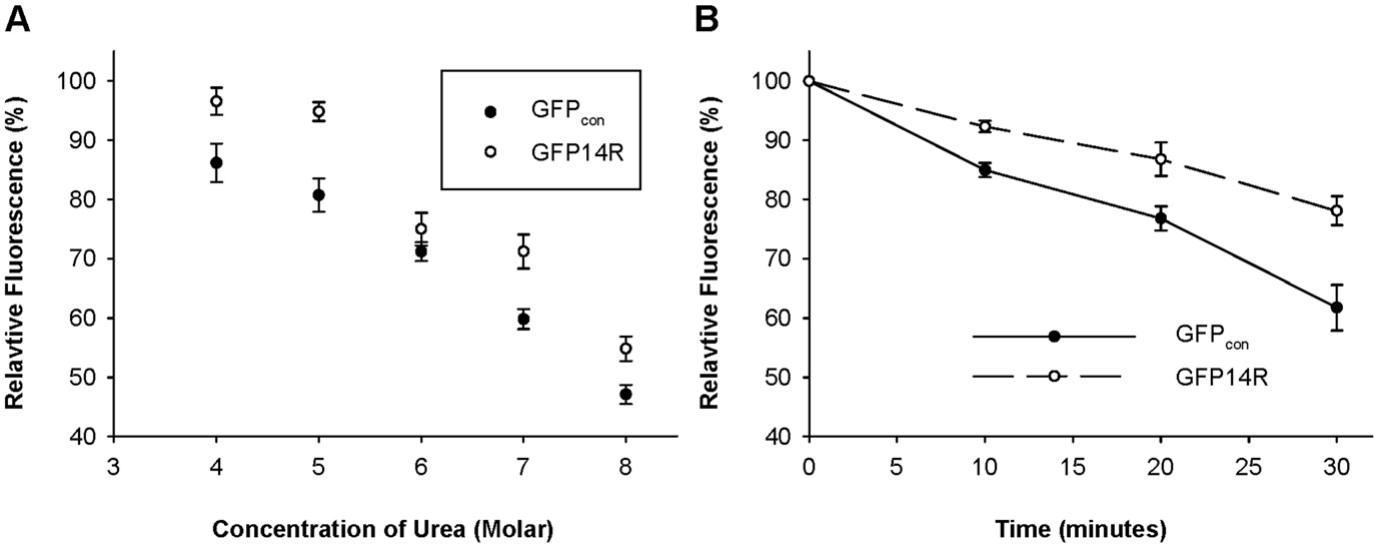
Effect of urea on the stability of the GFP variants. A) Protein samples were incubated at 50°C with different concentrations of urea from 4 M to 8 M for 30 minutes and the remaining fluorescence was measured. The fluorescence at time zero at the respective urea concentrations was taken into 100%. B) Protein samples were incubated at 50°C in presence of 6 M urea for different time intervals and the remaining fluorescence was measured. The fluorescence at time zero was taken into 100%. (Error bar – Standard deviation of the three independent experiments).

The stability of GFP14R variant against a chaotropic denaturant, urea, was tested. Purified samples of GFP14R and GFP_con_ were incubated with various concentrations of urea from 4 M to 8 M at 50°C for 30 minutes. The variant GFP14R showed stability to up to 5 M urea but began to lose its activity from 6 M urea. In contrast, GFP_con_ showed decreased activity from 4 M urea (Figure 4A). When a time-dependent assay was performed with 6 M urea at 50°C, the half-lives of GFP14R and GFP_con_ were estimated approximately to be 93 and 51 minutes, respectively (Figure 4B), which suggests that the stability of GFP against urea were positively affected by the mutagenesis of the surface lysines to arginines.

**Figure 5:**
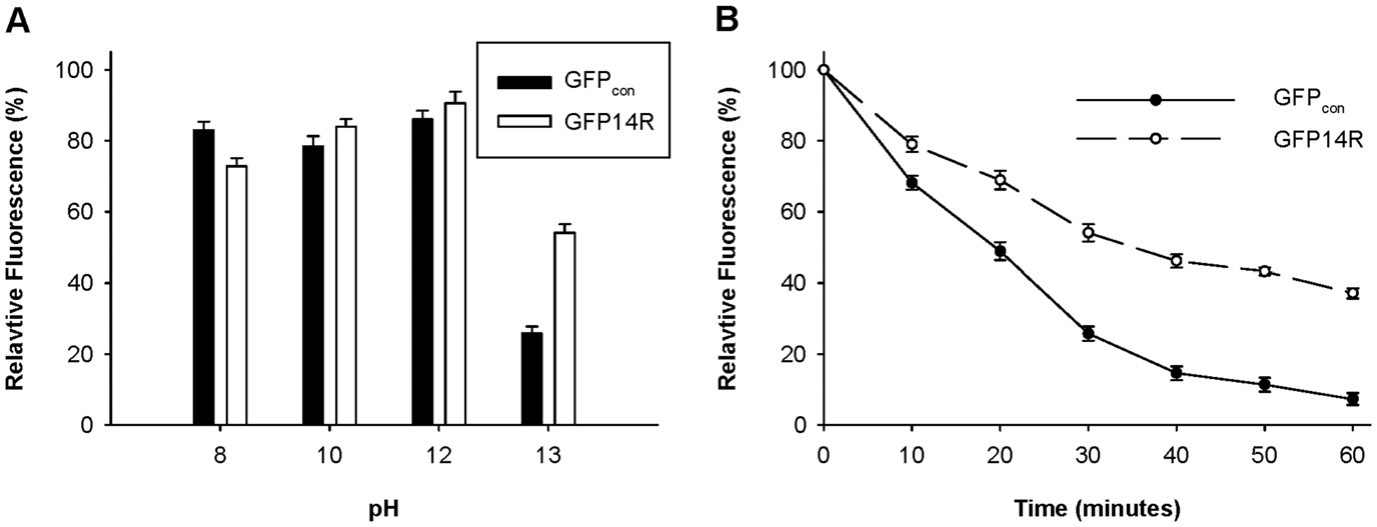
Effect of pH on the stability of the GFP variants. A) Protein samples were incubated at 60°C for 30 minutes in different pH buffer solutions and the remaining fluorescence was measured. The fluorescence at time zero at the respective buffer was taken into 100%. B) Protein samples in KCl buffer with pH 13.0 were incubated at 60°C for different time intervals and the remaining fluorescence was measured. The fluorescence at time zero was taken into 100%. (Error bar – Standard deviation of the three independent experiments).

The pKa values of the arginine and lysine are 12.48 and 10.53, respectively [32], which can lead to different stabilities of ionic interactions associated with the arginine residues and lysine residues under alkaline pH, as described in the introduction. To evaluate if such an effect can induce a difference in protein stability against alkaline pH, GFP_con_ and GFP14R were incubated at a range of pH, and their fluorescence after 30 minutes of incubation at 60°C was measured. The experiments revealed both GFP14R and GFP_con_ to be relatively stable up to pH 12.0, but the GFP14R showed higher stability than GFP_con_ at pH 13.0 (Figure 5A). The time-dependent assay at pH 13.0 at 60°C confirmed the GFP14R mutant to have higher stability than the control (Figure 5B). At pH 13.0, the half-lives of the GFP_con_ and GFP14R were 19 and 34 minutes, respectively. These results suggest that the introduced mutations stabilized the GFP protein to alkaline pH as expected.

**Figure 6:**
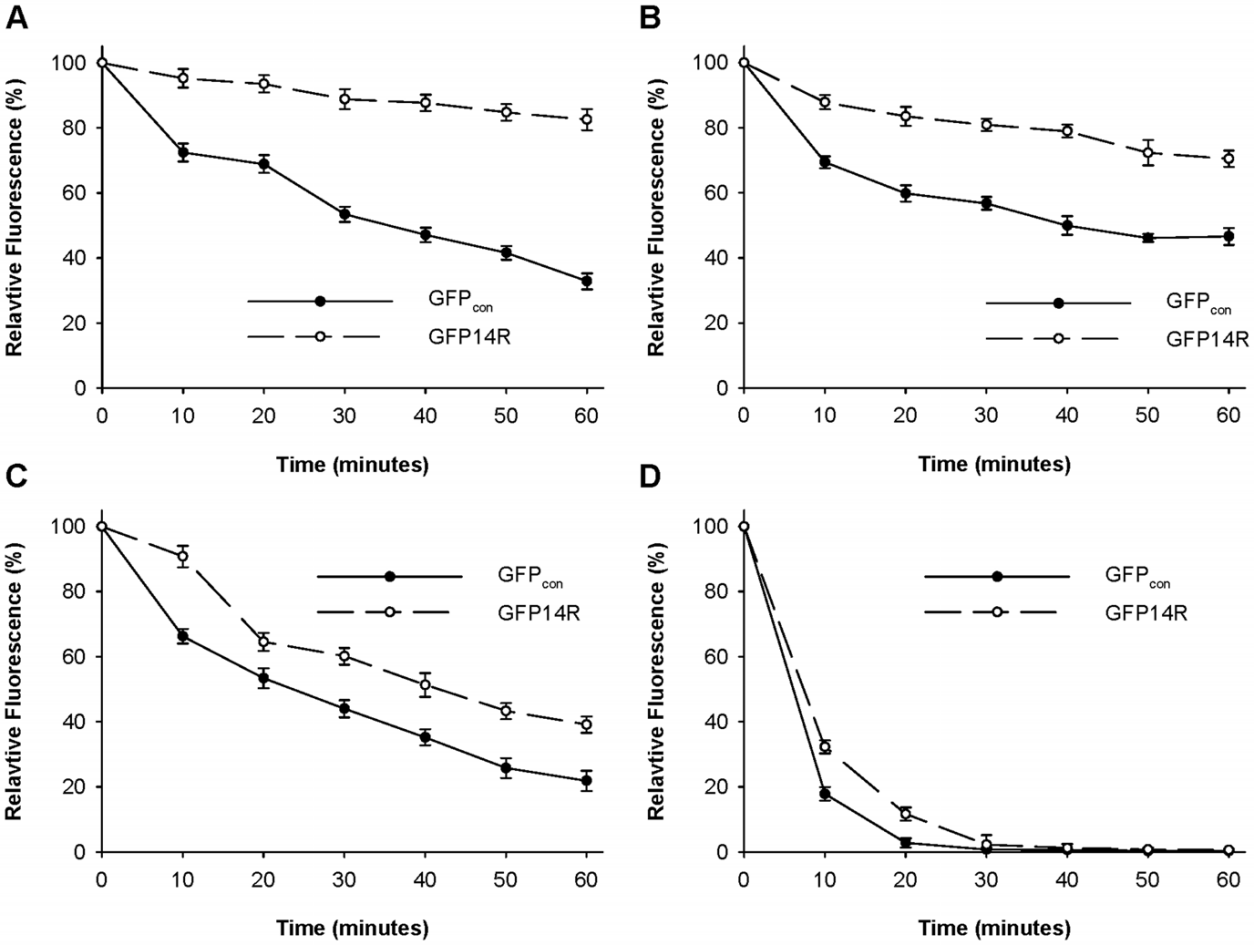
Effect of ionic detergents on the stability of the GFP variants. Protein samples were incubated with 1% SDS (A), 1% SDBS (B), 1% CTAB (C) and 1% DTAC (D) at 50°C for different time intervals and the remaining fluorescence was measured. The fluorescence at time zero at the respective detergent was taken into 100%. (Error bar – Standard deviation of the three independent experiments).

**Figure 7:**
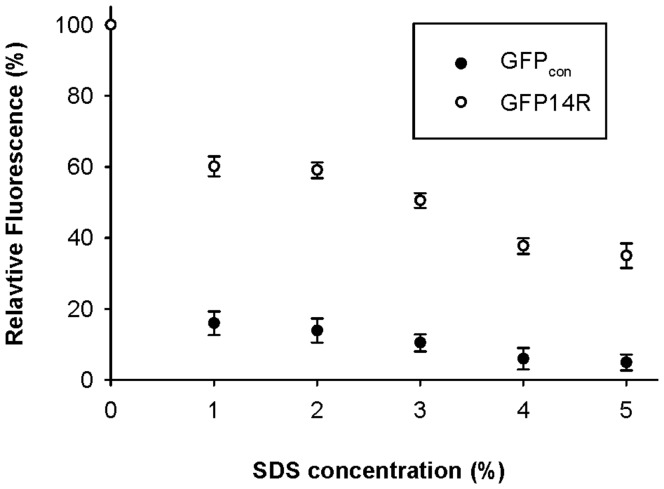
Effect of SDS concentration on the stability of the GFP variants. Protein samples were incubated with different concentrations of SDS at 50°C for 30 minutes and the remaining fluorescence was measured. The fluorescence at time zero at the respective SDS concentrations was taken into 100%. (Error bar – Standard deviation of the three independent experiments).

Two anionic detergents (sodium dodecyl sulphate (SDS) and sodium dodecyl benzene sulfonate (SDBS)) and two cationic detergents (cetyl trimethyl ammonium bromide (CTAB) and dodecyl trimethyl ammonium chloride (DTAC)) were tested to examine the effect of detergent on the stability of the GFP variant. GFP_con_ and GFP14R were incubated in 1% of the respective detergents, and a time-dependent fluorescence assay was performed. As shown in Figure 6, although the deactivation profiles were different according to the added detergents, the GFP14R always showed higher stability than GFP_con_. Table 2 lists the estimated half life (t_1/2_) of each variant in the presence of the ionic detergents. Interestingly, the stabilization effect was higher in anionic detergents than in cationic detergents. In particular, the effect was most marked in SDS. The stability of GFP14R was examined further in a range of SDS concentrations. The proteins were incubated with 1% to 5% SDS at 50°C for 30 minutes, and their remaining fluorescent activities were determined. As shown in Figure 7, GFP_con_ was mostly inactivated by the addition of SDS at all concentrations, but GFP14R showed approximately 50% of activity, even at 5% SDS. These results suggest that the variant GFP14R is more rigid and stable against detergents compared to GFP_con_.

### Analyses of Electrostatic Interactions of GFP14R with Respect to Lysine and Arginine

The above results confirmed that the introduction of arginines to the 14 Lys residues of GFP enhanced the GFP stability against urea, alkaline pH and detergents. Although the thermal stability of GFP was unaffected by mutagenesis, the results suggested the possibility of changes in the interactions associated with the surface basic residues toward protein stabilization. To check the possibility more directly, the changes in electrostatic interactions induced by the mutations was examined by analyzing the salt-bridges and hydrogen bonds in GFP14R. The analysis was also expected to answer one of the basic questions described in the introduction: can the replacement of arginines with lysines on the protein surface induce more electrostatic interactions in the protein?

As mentioned above, the similar spectral properties of GFP_con_ and GFP14R (Figure 2) suggest that the essential structure of GFP might not be changed significantly by the mutations. This prompted us to predict the three dimensional structure of the GFP14R by energy minimization method. The GFP crystal structure with PDB Id 1GFL [29] was used as a template structure in the modeling, which allowed us to assume that the local energy minimum of the modeled GFP variant was its native state. The structure of GFP_con_ was also modeled using the same procedure as a control. The *in silico* models of GFP14R and GFP_con_ did not show significant deviations from the template structure with a root mean square deviation of approximately 0.1 Å. The molecular dynamic simulations for the modeled structures showed that the root mean square deviation (rmsd) for the GFP_con_ and GFP14R were found to be within 2.5 Å throughout the 10 nanosecond simulation (Figure S4), which confirmed that the predicted models were substantially stable. In the analyses of the electrostatic interactions of the two modeled proteins, the distance between N-O for an electrostatic pair to form a strong salt-bridge interaction was considered to be ≤4 Å, and a hydrogen bond was determined using the criteria of approximately 2.5 to 3.2 Å [16], [33].

Table 3 lists the salt-bridges and hydrogen bonds associated with the 19 surface Lys residues in the modeled GFP_con_ as well as the altered interactions in the modeled GFP14R for the 19 residues. The energy minimization process in the modeling of GFP_con_ predicted three new salt bridge interactions (K79-E5, K131-D103, and K166-D180) and 12 new hydrogen bonds compared to the interactions in the original GFP structure shown in Table 1, but 8 hydrogen bonds were lost, while retaining the salt bridges. This resulted in 11 salt bridges and 30 hydrogen bonds associated with the 19 surface lysines of GFP_con_. On the other hand, in the model of GFP14R, 16 salt bridges and 39 hydrogen bonds were found to be involved in the 19 basic residues. This suggests that the mutagenesis of the surface 14 lysines to arginines induced the addition of 5 more salt bridges and 9 hydrogen bonds as a net result. A comparison of the 11 salt-bridges in GFP_con_ and 16 salt bridges in GFP14R showed that 9 salt-bridges of GFP_con_ were maintained, 2 salt-bridges of GFP_con_ were lost, and 7 salt-bridges were newly formed by mutagenesis. For the hydrogen bonds, 24 hydrogen bonds of GFP_con_ were maintained, 6 were lost, and 15 new hydrogen bonds were generated. This clearly shows that the introduction of arginines in the 14 surface lysine residues modified the electrostatic interactions in GFP significantly. This also demonstrates that arginine is more favorable to form salt bridges and hydrogen bonds on the protein surface than lysine. The increased numbers of electrostatic interaction of GFP14R compared to GFP_con_ might be one of the factors affecting the stability improvement, although precisely how much the added salt-bridges and hydrogen bonds induced by the mutagenesis contributed to the stability enhancement of GFP is difficult to determine because other factors, such as electrostatic interactions between the basic residues and water molecules, could also affect protein stability.

**Table 2:** Half life of the GFP variants in different ionic detergents.

	t_1/2_ (minutes)	
Denaturant	GFP_con_	GFP14R
1% SDS	37.44±2.27	166.79±3.98
1% SDBS	27.04±1.24	78.73±9.2
1% CTAB	22.18±1.2	31.84±1.7
1% DTAC	3.83±0.32	6.45±0.3

SDS - sodium dodecyl sulphate.

SDBS - sodium dodecyl benzene sulfonate.

CTAB - cetyl trimethyl ammonium bromide.

DTAC - dodecyl trimethyl ammonium chloride.

Error range – Standard error calculated from three independent experiments.

**Table 3:** Comparison of salt-bridge and hydrogen bonds interactions of the GFP variants.

		GFP_con_		GFP14R		
Lysine	Secondary structure	Salt-bridge	Hydrogen bond	K to R	Salt-bridge	Hydrogen bond
3	Loop			+	E6*	E6*
26	β-strand 2	D21	D21, G24	+	D21	D21
41	β-strand 3		D36, T43	−	D36*	D36
45	β-strand 3	E213, D210	E32, E213, D210	−	E213, D210	E32, D210, E213
52	Loop			+	D216*	T50*, D216*
79	Loop	E5	E5, P75	+	E5, D76*	E5, Y74*, P75, D76*
101	Loop	D102	D102, Q177, L178	+	D102	D103*, Q177, L178
107	β-strand 5		N105, K126	−		N105, K126
113	β-strand 5	E111, E115	E111, V120	−	E111, E115	E111, E115*, V120
126	β-strand 6	D21	D21	−	D21	D21, G127*
131	Loop	D103	D102, D103, D134	+	D133*	D102, D103, D134
140	Loop		D133, G134, N135	+	D173*	D133, G134, N135, E172*, D173*
156	Loop			+		N159*
158	Loop	D155	D155, Q184	+		D155, Q157*
162	β-strand 8		M153	+		M153, N164*
166	β-strand 8	D180	D180	+	D180	D180
209	Loop		Y143, H217	+	D216*	D216*, H217
214	Loop		N212	+		E213*
238	Loop			+		

‘+’ – indicates the presence of mutation of lysine to arginine.

‘−’ – indicates the absence of mutation of lysine to arginine.

‘*’– new interactions.

### Structural Analysis of the Newly Formed Salt-bridges in GFP14R

Above study on the electrostatic interactions in GFP14R revealed that the addition of arginines in the lysine residues generated salt-bridges and hydrogen bonds more favorably. This is correlated with the different geometric structure of the basic side chains of arginine and lysine described in the introduction. The newly formed 7 salt-bridges by lysine to arginine mutation were examined structurally using their modeled structures to identify more directly the difference between lysine and arginine in the formation of electrostatic interactions.

**Figure 8:**
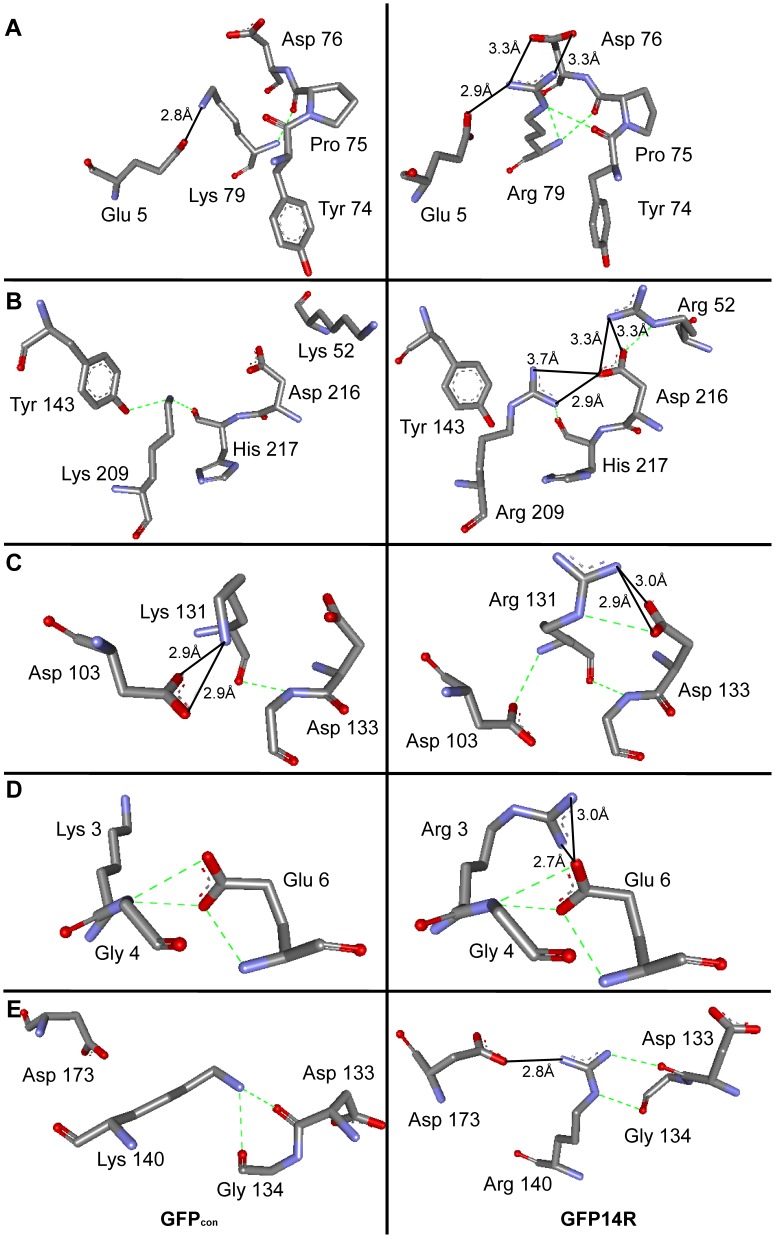
Structural representation of the new salt-bridge interactions. Comparison of salt-bridge interacting distances between GFP_con_ (left panel) and GFP14R (right panel) of (A) Lys/Arg79-Glu5, (B) Lys/Arg209-Asp216 and Lys/Arg52-Asp216, (C) Lys/Arg131-Asp103, (D) Lys/Arg3-Glu6, and (E) Lys/Arg140- Asp173. Black straight lines and green dashed lines indicate the salt-bridge and hydrogen bond interactions, respectively. Graphical image was generated using Discovery Studio Visualizer from Accelrys Software Inc.

In GFP_con_, Lys79 formed a salt bridge with Glu5, whereas arginine at the same position in GFP14R exhibited three different interactions (Figure 8A): two salt-bridges with Glu5 and Asp76 using N^η1^ and N^η2^, and a hydrogen bond using N^ε^ with the backbone carbonyl oxygen of Tyr74. This change in side chain at the 79^th^ position also favored its backbone nitrogen to form hydrogen bonds with the backbones of Pro75 and Asp76. Lys209 and Lys52 of GFP_con_ could not produce any salt-bridge interaction, even though an appositely charged residue, Asp216, was available around them. On the other hand, when arginine was introduced in these two positions, both Arg209 and Arg52 formed salt bridge interactions with Asp216 (Figure 8B). The loss of a hydrogen bond between Arg209 and Tyr143 in GFP14R, which was present in GFP_con_, might be compensated for by the new salt bridge. The salt bridge of Lys131-Asp103 in GFP_con_ was lost in GFP14R, but Arg131 formed a salt-bridge with Asp133 (Figure 8C). The salt bridge with Asp103 was lost because the N^ε^ of Arg131 favored a hydrogen bond, whereas N^η1^ and N^η2^ favored salt-bridge with Asp133. The interaction with Asp103 was maintained by a hydrogen bond with its backbone. The surface exposed Arg3 in GFP14R located at the flexible N-terminus of the protein showed the possibility of a new interaction by making a salt-bridge with Glu6 (Figure 8D). The side chain of Lys140 in GFP_con_ made two hydrogen bonds with the backbone of Asp133 and Gly134. On the other hand, the guanidino moiety of arginine at this position in GFP14R formed a new salt-bridge with Asp173 using N^η1^ whilst retaining the hydrogen bonds using N^ε^ with Gly134 and N^η2^ with Asp133 (Figure 8E).

The newly predicted salt-bridges in GFP14R were ≤4 Å whereas they were ≥4 Å for the GFP_con_ at the seven positions. Six new salt-bridge interactions in GFP14R were formed in the loop regions of the protein except for the salt-bridge of K41-D36 where the variant does not contain a mutation at K41. Arginine adopts larger number of conformations due to its size, side-chain flexibility and more atoms capable of carrying charges than lysine [16]. Both the loop flexibility and the geometric advantage of arginine were presumed to increase the probability of more electrostatic interactions in GFP14R.

The distances between the atoms involved in the new ionic pairs of GFP14R were further analyzed by performing molecular dynamics simulation at room temperature to increase the fidelity of the newly formed salt-bridges. Two (R52-D216 and R140-D173) salt-bridges predicted using the modeled structure of the GFP14R were found to have lower distance variation during the 10 nanoseconds simulation than the GFP_con_ while the distance between the atoms for the remaining salt-bridges were found to be similar to the GFP_con_ (Figure S5). These dynamics simulation results suggest the possibility that the predicted salt-bridges using the static modeled structures were not consistent for all the cases.

## Discussion

The effects of mutating surface lysine of GFP to arginine on the protein stability and structure were studied. The GFP14R variant was generated by replacing 14 surface lysines of native GFP with arginine and by retaining 5 surface lysines involved in the formation of the GFP superstable core structure. The variant showed a low folding rate (Figure S6), but its final structure was presumed to be relatively unchanged. Stability tests of the variant showed that the mutagenesis of surface lysine to arginine was effective in enhancing the stability of GFP against urea, alkaline pH and detergents, but did not affect the thermal stability of GFP. Structural analyses of the electrostatic interactions confirmed not only that additional salt-bridges and hydrogen bonds had been generated by the mutations, but also the geometric properties of the guanidinium group in Arg had such effects. These results showed the following: 1) the introduction of arginines on the surface lysine residues can stabilize a protein, but the effect can differ according to the denaturation conditions; 2) the mutagenesis of surface lysine to arginine can induce changes in the electrostatic interactions in an additional manner, which might be a factor in enhancing the stability; and 3) such mutagenesis can affect the protein folding unfavorably.

Interestingly, the thermal stability of GFP was not changed by mutagenesis, whereas the stability against urea, alkaline pH and detergents were improved. One explanation for this might be the difference in the mechanism between thermal denaturation and other chemical-mediated denaturations. In the case of thermal denaturation, the energy given to the protein induces the overall movement of the atoms in a protein, which leads to a nonspecific and simultaneous disruption of various interactions for protein folding. Therefore, the enhanced electrostatic interactions caused by mutagenesis might not so effective in preventing thermal denaturation in the detection range. In addition, the interactions formed on the protein surface, as in this case, contribute only slightly to protein thermal stability [34], which may enhance such effect more. On the other hand, urea, alkaline pH and ionic detergents begin to denature proteins by interacting with the specific atoms involved in electrostatic interactions in a protein. Therefore, the lysines and arginines generally involved in electrostatic interactions might be affected more specifically and sensitively by the added chemical denaturants. Of course, further studies will be needed to understand the effect more precisely.

In the study on the pH effect, GFP14R showed higher stability than GFP_con_ at pH 13.0. A theoretical estimation of the protonated states of the basic residues of Arg and Lys based on their side chain pKa’s showed that 20% of the arginine side chains were protonated at pH 13.0, whereas most of the Lys side chains are deprotonated at this pH (Figure S7). This may lead to the difference in the ionic interactions on the surface of the two GFP proteins and explain the lower stability of the GFP_con_ than GFP14R at the alkaline pH. On the other hand, GFP_con_ was relatively stable at pH12 (Figure 5A), despite the almost complete deprotonation of the Lys basic residue at this pH (Figure S7), which suggests there is no direct correlation between the ionic states of the Lys residues estimated theoretically and the GFP stability. The difference between environmental pH on the protein surface and solvent bulk pH might lead to this kind of result. In addition, hydrophilic amino acids other than Arg and Lys can also be affected by the pH, which might also induce such an effect.

A study of the ionic detergent effect on GFP stability showed that negative charged detergents, such as SDS, had a more prominent effect than the positive charged detergent. This may be explained by considering the mechanism of the protein unfolding caused by the detergent. Although the precise mechanism is unclear, it has been suggested that protein unfolding by ionic detergents is a combination of hydrophobic and electrostatic interactions [35]–[37]. A previous study on the SDS-induced protein denaturation showed that SDS binds to the polypeptide chain initially by a hydrophobic interaction followed by charge-charge repulsion between the SDS micelles and anionic side chains of the protein as the SDS concentration is increased [38]–[40]. By surface Lys to Arg mutagenesis, the frequency of arginine was increased from 2.5% to 8.4% for GFP14R, resulting in an increasingly positively charged environment on the protein surface. Therefore, the negatively charged hydrophilic head of the anionic detergent might bind to these positively charged arginine side chains more favorably in GFP14R, thereby reducing the possibility of charge-charge repulsion required for further denaturation. This could be an additional factor for the enhancement of GFP stability against anionic detergents in addition to the effect of increased electrostatic interactions in the protein, which might lead to the more prominent effect for anionic detergents.

In the analyses of electrostatic interactions of GFP14R using energy minimized model, it was predicted that 7 new salt-bridges were formed by the addition of arginines in the 6 lysine residues (K3, K52, K79, K131, K140 and K209) of GFP_con_. To evaluate how much the newly formed salt-bridges contribute to the stability of the GFP14R, a variant GFP6R, having arginines instead of lysines in these six positions, was generated and its stability was compared with GFPcon and GFP14R. The stability of the GFP6R was tested against the denaturing conditions such as 1% SDS and alkaline pH 13.0, the conditions in which the GFP14R showed predominantly higher stability. Interestingly, the stability of the GFP6R was not improved and similar to the control GFP (Figure S8). These results imply that the predicted new salt-bridges are not the effective stabilization factor for the enhanced stability of the GFP14R. According to the molecular dynamics study on the distance variations of the ion-pairs (Figure S5), there is a possibility that only two salt-bridges are effective among the 6 salt-bridges, which supports this experimental result. We presume that the enhanced stability of GFP14R was caused by the collective effects of the new electrostatic interactions including amino acid-water interactions as well as amino acid-amino acid interactions generated by the newly introduced 14 arginines. Further detailed study may be needed to explain this kind of collective effects.

From the expression study of GFP14R, the GFP protein was produced mainly in insoluble form and the productivity of soluble form was low. The folding rate of the GFP14R was confirmed to be low compared to GFP_con_ (Figure S6). On the other hand, the GFP14R showed higher stability for the chemical denaturants and comparable thermal stability compared to GFP_con_. These results indicate that the mutations introduced on the surface adversely affected protein folding, but the protein was stable once folded. This implies that the changed electrostatic interactions induced by the mutagenesis stabilized the GFP kinetically rather than thermodynamically. Further thermodynamic and kinetic studies may be needed to understand the stabilization mechanism more precisely.

The mutagenesis strategy in the present study still has limitations to be applied directly to protein stabilization. For example, the folding rate of the protein was reduced significantly by mutagenesis, which can cause a problem of target protein production. In addition, only GFP was tested as a target protein. The mutagenesis effect might differ according to the properties of the target proteins. Many factors, such as a protein’s original stability, protein structure, and the number and location of the surface lysine residues, can affect the folding efficiency and stability of the target proteins differently by the mutagenesis. Therefore, there is a need to study these effects further using other proteins. Nevertheless, this study is meaningful in that the effect of Lys and Arg on protein stability was studied more directly compared to other studies. This study highlights the possibility that the surface lysine mutagenesis to arginines can be considered one of the parameters in protein stability engineering.

## Materials and Methods

### Materials

The host bacterium *E. coli* strain DH5α was used for plasmid DNA preparation. The *E. coli* strain BL21 (DE3) was used as the expression host for production of the recombinant GFP variants. PCR reagents and T4 DNA ligase were purchased from New England Biolabs. Restriction Endonucleases were obtained from Fermentas. IPTG (isopropyl-D-thiogalactopyranoside), sodium dodecyl sulphate (SDS), sodium dodecyl benzene sulfonate (SDBS), cetyl trimethyl ammonium bromide (CTAB), dodecyl trimethyl ammonium chloride (DTAC) and other chemicals were purchased from Sigma chemicals (St. Louis, MO, USA). The Ni-NTA affinity column HisTrapHP was supplied by GE healthcare (Sweden). The pET30b vector was obtained from Novagen.

### Construction of Plasmid

The genes for GFP_con_, GFP19R, GFP14R and GFP6R were synthesized by Genscript Corporation (New Jersey, USA). The synthesized genes were cloned into the NdeI and XhoI restriction sites of the pET30b vector with the hexa histidine tag at the C-terminal.

### Expression and Purification of GFP Variants

The GFP variants cloned into the pET30b vector were transformed to the BL21 (DE3) strain for expression. The proteins were expressed at 37°C for five hours unless mentioned otherwise after induction with 1 mM IPTG at an O.D. of 0.6–0.8. The cell lysate was prepared from a cell pellet corresponding to 1 ml of 3 O.D. cells using a BugBuster protein extraction kit (Novagen) and centrifuged at 12000 rpm for 30 minutes to separate the soluble and insoluble fraction for SDS-PAGE analysis. The variant GFP14R was expressed at 25°C for 20 hours to increase the protein yield. The cells were harvested by centrifugation at 5000 rpm for 15 minutes and resuspended with PBS buffer (Phosphate Buffered Saline buffer −137 mM NaCl, 2.7 mM KCl, 10 mM Na_2_HPO_4_, 2 mM KH_2_PO_4_, and pH 7.4). The cells were disrupted using a French Press and centrifuged at 20000 rpm for 30 minutes at 4°C to collect the soluble fraction. The soluble proteins were then purified using 5 ml volume Ni-NTA column equilibrated with 50 mM sodium phosphate buffer (50 mM Sodium Phosphate prepared from 1 M Na_2_HPO_4_ and 1 M NaH_2_PO_4_, 300 mM NaCl, pH 7.4) containing 10 mM imidazole. The column was then washed with 5 column volumes of 50 mM sodium phosphate buffer containing 50 mM imidazole and the elution was done using 50 mM sodium phosphate buffer containing 500 mM imidazole. The elution fractions were analyzed by SDS-PAGE, and the fractions enriched with recombinant proteins were collected and dialyzed with PBS buffer pH 7.4. The concentrations of the purified protein samples were determined using a UV/VIS spectrophotometer, at 280 nm using the extinction coefficient calculated from the protein sequences.

### Fluorescence Measurement

The whole cell fluorescence of the GFP variants was measured using Perkin Elmer/Wallac Victor 2 Multilabel Counter (1420-011) with excitation and emission at 485 nm and 515 nm respectively, from 5.0 nm slits after resuspending the cell pellet in PBS buffer. The excitation and emission spectrum of the purified 5 µM protein samples were measured using a Perkin Elmer LS-55 spectrofluorimeter with excitation/emission slits of 2.5 nm. Unless mentioned otherwise, fluorescence was measured with 5 µM protein samples using 96 well plates with Perkin Elmer/Wallac Victor 2 Multilabel Counter for all the stability assays.

### Thermal Stability of the GFP Variants

A temperature-dependent assay was performed by incubating the samples from 40°C to 100°C at 10°C intervals for 30 minutes and the fluorescence was measured at room temperature. A time-dependent assay was performed by incubating the samples at 70°C up to 30 minutes at 10 minute intervals and the fluorescence was measured at room temperature.

### Stability of the GFP Variants at Different pH

Protein samples were incubated with a range of buffers containing pH 8.0 (50 mM Tris-HCl), pH 10.0 (50 mM Sodium Carbonate), pH 12.0 and pH 13.0 (50 mM KCl) at 60°C for 30 minutes and the fluorescence was measured at room temperature. In addition, a time-dependent assay was performed by incubating the samples with 50 mM KCl (pH 13.0) at 60°C up to 60 minutes at 10 minutes intervals and the fluorescence was measured at room temperature.

### Stability of the GFP Variants Against Urea and Ionic Detergents

To determine the stability of the variants against chemical denaturants, the samples were incubated with 6 M urea at 50°C for different time intervals up to 30 minutes and the fluorescence was measured at room temperature. The stability of the variants were also measured in presence of ionic detergents such as sodium dodecyl sulphate (SDS) and sodium dodecyl benzene sulfonate (SDBS) which are anionic and cetyl trimethyl ammonium bromide (CTAB) and dodecyl trimethyl ammonium chloride (DTAC) which are cationic. For this, the samples were incubated with 1% of the detergent at 50°C for different time intervals up to 60 minutes. In addition, stability of the variants was measured at different SDS concentrations starting from 1% to 5% by incubating the samples at 50°C for 30 minutes. In all the above experiments, the pH of the protein samples was maintained at 7.4 with a PBS buffer.

### Refolding of the GFP Variants

The folding efficiency of the GFP variants were evaluated by denaturing the protein samples at harsh conditions and allowed to refold after dilution. 25 µM protein samples were denatured by incubating at 95°C for 5 minutes in PBS buffer containing 8 M urea and 5 mM dithiothreitol (DTT). The denatured samples were diluted to 100 fold using PBS buffer containing 5 mM DDT and the fluorescence was measured at room temperature for 30 minutes with 3 seconds time interval using Perkin Elmer LS-55 spectrofluorimeter (490 nm excitation, 511 nm emission, 2.5 nm excitation/emission slit). The recovered fluorescence was normalized to 1 by dividing with maximum fluorescence value of the spectra.

### Modeling and Molecular Dynamics Simulation of the GFP Variants

The three dimensional structure of GFP_con_ and the variant were modeled using the GFP structure with PDB Id 1GFL as the template [29]. The models were created using the Build Homology Models protocol from the Protein Modeling module of the Discovery Studio 2.0 (Accelrys Software Inc., San Diego, CA, USA). Energy minimization was performed using the minimization protocol from the simulation module of the Discovery Studio 2.0 according to the conjugate gradient method after typing with the automatic assignment of the Momany & Rone CHARMm force field and the non-bonded interactions cutoff set to 10 to 12 Å. The solvent conditions used were distance-dependent dielectrics with a dielectric constant set to 4 to mimic the screening effects of the solvent at the crude level. The energy minimization cycles were carried out for 3000 steps until the RMS gradient tolerance was ≤0.1 kcal mol^−1^Å^−1^. The *in silico* models of the GFP variants showed only slight deviation from the template structure with a root mean square deviation of approximately 0.1 Å. The salt-bridge and hydrogen bond interactions were analyzed using Discovery Studio 2.0 (Accelrys Software Inc., San Diego, CA, USA). The predicted GFP variants generated above by molecular modeling were adopted as the starting structure for performing molecular dynamics (MD) simulation using the MD simulation package GROMACS 4.5.5 [41]. Using GROMOS96 53a6 force field, the structure was centered in a cubic unit cell with 1.0 nm from the box edge and solvated using SPC/E water model. The system was then neutralized with Na^+^ and Cl^−^ as counter ions and energy minimized using the steepest descent algorithm for 2000 steps. The energy minimized system was equilibrated using the position restrained simulation under an *NVT* ensemble (constant Number of particles, Volume and Temperature) for 100 picoseconds to stabilize the temperature at 300 K with Berendsen thermostat followed by an *NPT* ensemble (constant Number of particles, Pressure and Temperature) for 100 picoseconds to stabilize the pressure at 1.0 bar with Parrinello-Rahman pressure coupling factor. Finally unrestrained MD simulation was performed for 10 nanoseconds with Berendsen thermostat of 300 K and the pressure at 1.0 bar with Parrinello-Rahman pressure coupling factor. The trjconv, g_rms, make_ndx and g_dist utilities of GROMACS 4.5.5 were used to analyze the MD results.

## Supporting Information

Figure S1 **SDS-PAGE gel shows the protein expression profile.** A) Lane 1 and 2 indicate the soluble fraction of GFP_con_ and GFP19R, respectively, and lane 4 and 5 indicate the insoluble fraction of GFP_con_ and GFP19R, respectively, induced with 1 mM IPTG at 37°C for 5 hours. Lane 3 and 6 indicate the soluble and insoluble fraction of the GFP19R, respectively, induced with 1 mM IPTG at 25°C for 5 hours. B) Lane 1 and 2 indicate the soluble fraction of GFP_con_ and GFF14R, respectively and lane 3 indicates the insoluble fraction of GFP14R, induced with 1 mM IPTG at 37°C for 5 hours. Lane M - protein marker.
(TIF)

Figure S2 A) SDS-PAGE gel shows the protein expression profile of the GFP_con_ and GFP14R induced with 1 mM IPTG at 25°C for 5 hours. Lane 1 and 2 indicate the soluble fraction of GFP_con_ and GFF14R, respectively and the lane 3 and 4 indicate the insoluble fraction of GFP_con_ and GFF14R, respectively. Lane 5 and 6 show the purified protein of GFP_con_ and GFP14R respectively. Lane M - protein marker. B) Whole cell fluorescence of GFP_con_ and GFP14R normalized by the absorbance at 600 nm. (au – arbitrary units).
(TIF)

Figure S3 **Relative specific fluorescence was measured using 2** **µM of the purified protein samples for the GFP_con_ and GFP14R.** (au – arbitrary units).
(TIF)

Figure S4 **The main chain rmsd (root mean square deviation) of the GFP_con_ and GFP14R over the 10 nanoseconds simulation.** (nm – nanometer, ps – picoseconds).
(TIF)

Figure S5 **The rmsd (root mean square deviation) distance between the ionic pair atoms over the 10 nanoseconds simulation.** (nm – nanometer, ps – picoseconds).
(TIF)

Figure S6 **Denaturation and refolding of the GFP variants.** The folding efficiency of the GFP variants were measured by denaturing in 8 M urea at 95°C and followed by renaturation by dilution at room temperature. Normalized fluorescence in arbitrary units (au) was plotted against time.
(TIF)

Figure S7 **Protonation states were estimated theoretically using the equation log([AH]/[A^−^])  =  pKa-pH.** The side chain pKa values of lysine (pKa 10.53) and arginine (pKa 12.48) were used to estimate the ratio of [AH] over [A^-^] for each pH and converted to percentage.
(TIF)

Figure S8 **A)** Stability of the GFP variants in presence of 1% SDS at 50°C for 30 minutes. The fluorescence at time zero in 1% SDS was taken into 100%. **B)** Stability of the GFP variants in presence of 50 mM KCl buffer pH 13.0 at 60°C for 30 minutes. The fluorescence at time zero at pH 13.0 was taken into 100%. (Error bar – Standard deviation of the three independent experiments).
(TIF)
