# Supplementary figures and images for: A Study on the Effect of Surface Lysine to Arginine Mutagenesis on Protein Stability and Structure Using Green Fluorescent Protein
Source: PLoS One. 2012 Jul 9;7(7):e40410. doi: 10.1371/journal.pone.0040410 (PMC3392243; doi:10.1371/journal.pone.0040410)

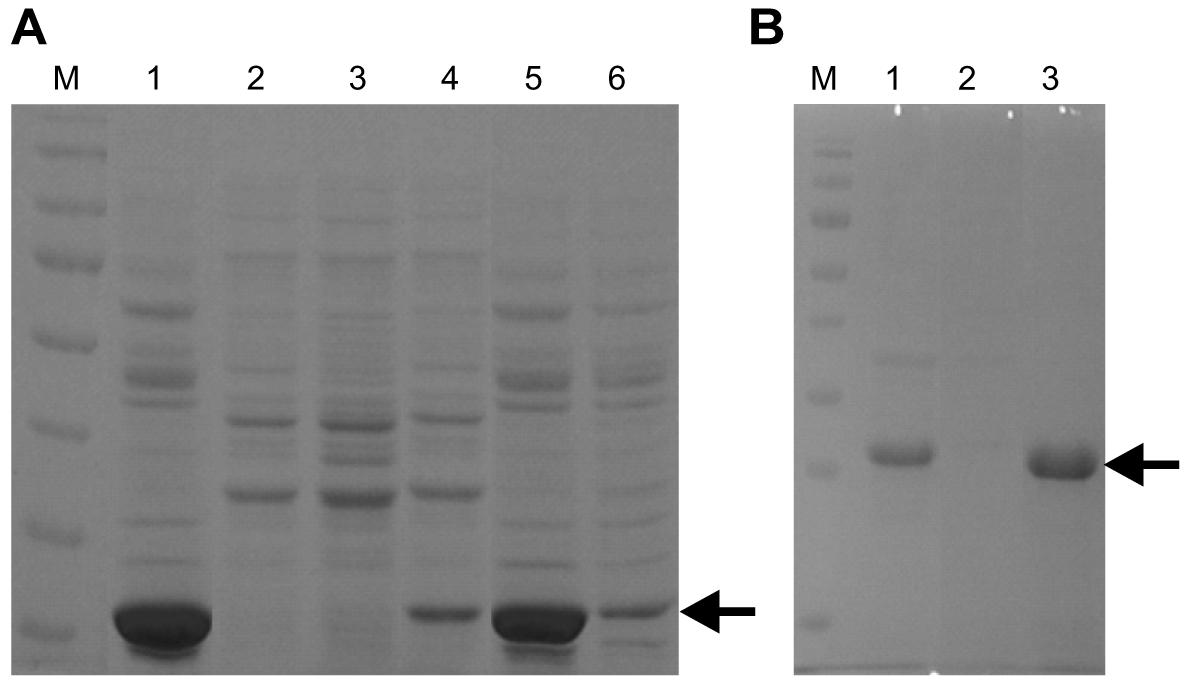

Supplement: Figure S1 — SDS-PAGE gel shows the protein expression profile. A) Lane 1 and 2 indicate the soluble fraction of GFPcon and GFP19R, respectively, and lane 4 and 5 indicate the insoluble fraction of GFPcon and GFP19R, respectively, induced with 1 mM IPTG at 37°C for 5 hours. Lane 3 and 6 indicate the soluble and insoluble fraction of the GFP19R, respectively, induced with 1 mM IPTG at 25°C for 5 hours. B) Lane 1 and 2 indicate the soluble fraction of GFPcon and GFF14R, respectively and lane 3 indicates the insoluble fraction of GFP14R, induced with 1 mM IPTG at 37°C for 5 hours. Lane M - protein marker. (TIF) [file pone.0040410.s001.tif]

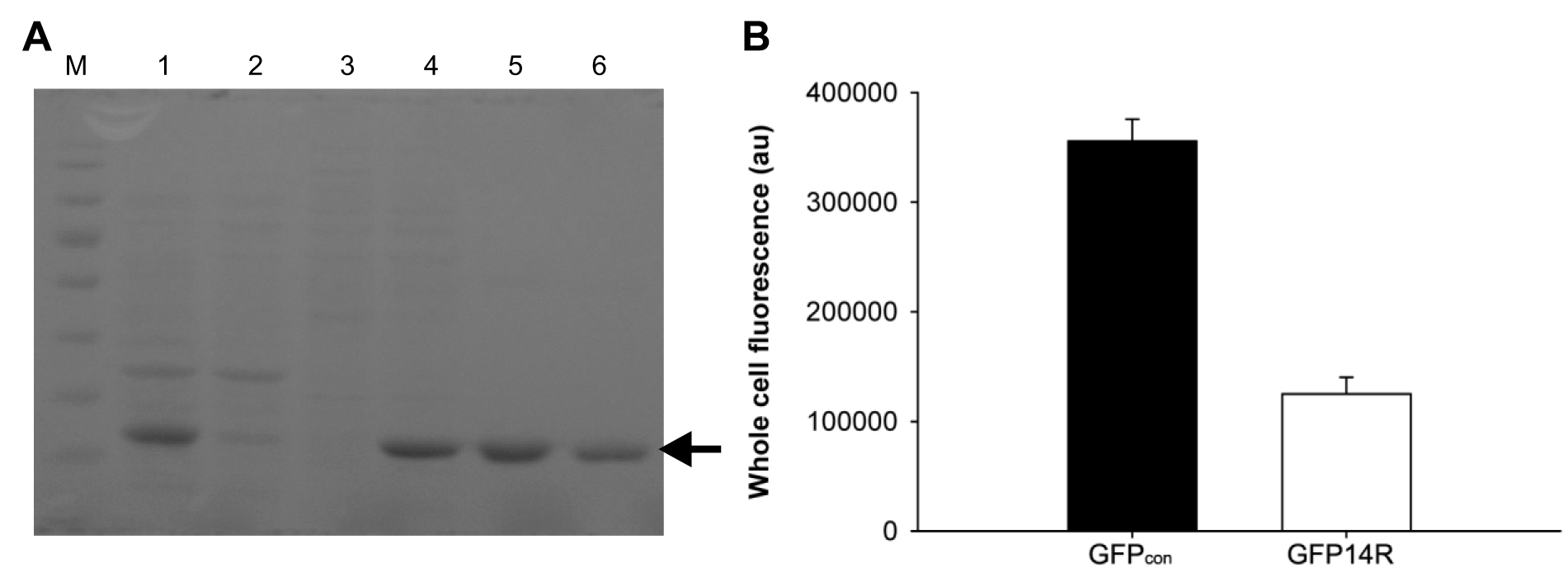

Supplement: Figure S2 — A) SDS-PAGE gel shows the protein expression profile of the GFPcon and GFP14R induced with 1 mM IPTG at 25°C for 5 hours. Lane 1 and 2 indicate the soluble fraction of GFPcon and GFF14R, respectively and the lane 3 and 4 indicate the insoluble fraction of GFPcon and GFF14R, respectively. Lane 5 and 6 show the purified protein of GFPcon and GFP14R respectively. Lane M - protein marker. B) Whole cell fluorescence of GFPcon and GFP14R normalized by the absorbance at 600 nm. (au – arbitrary units). (TIF) [file pone.0040410.s002.tif]

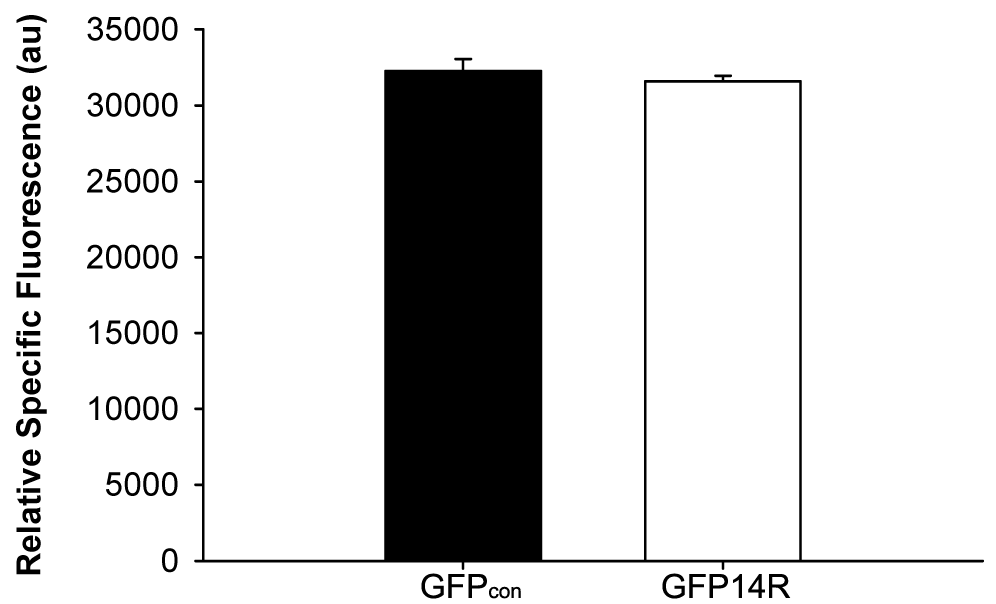

Supplement: Figure S3 — Relative specific fluorescence was measured using 2 µM of the purified protein samples for the GFPcon and GFP14R. (au – arbitrary units). (TIF) [file pone.0040410.s003.tif]

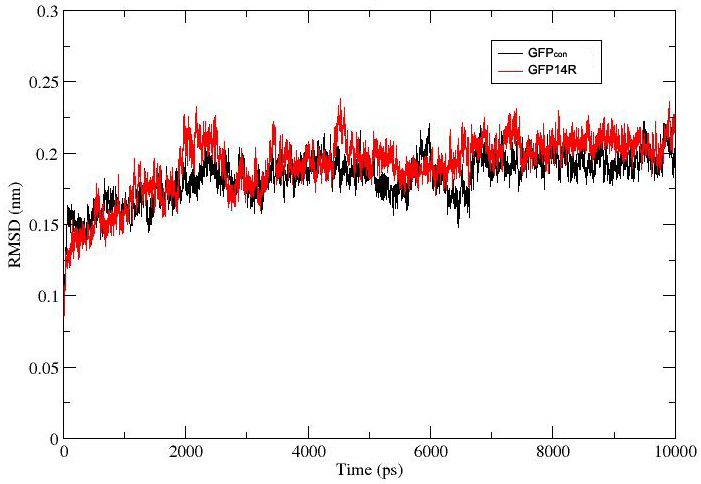

Supplement: Figure S4 — The main chain rmsd (root mean square deviation) of the GFPcon and GFP14R over the 10 nanoseconds simulation. (nm – nanometer, ps – picoseconds). (TIF) [file pone.0040410.s004.tif]

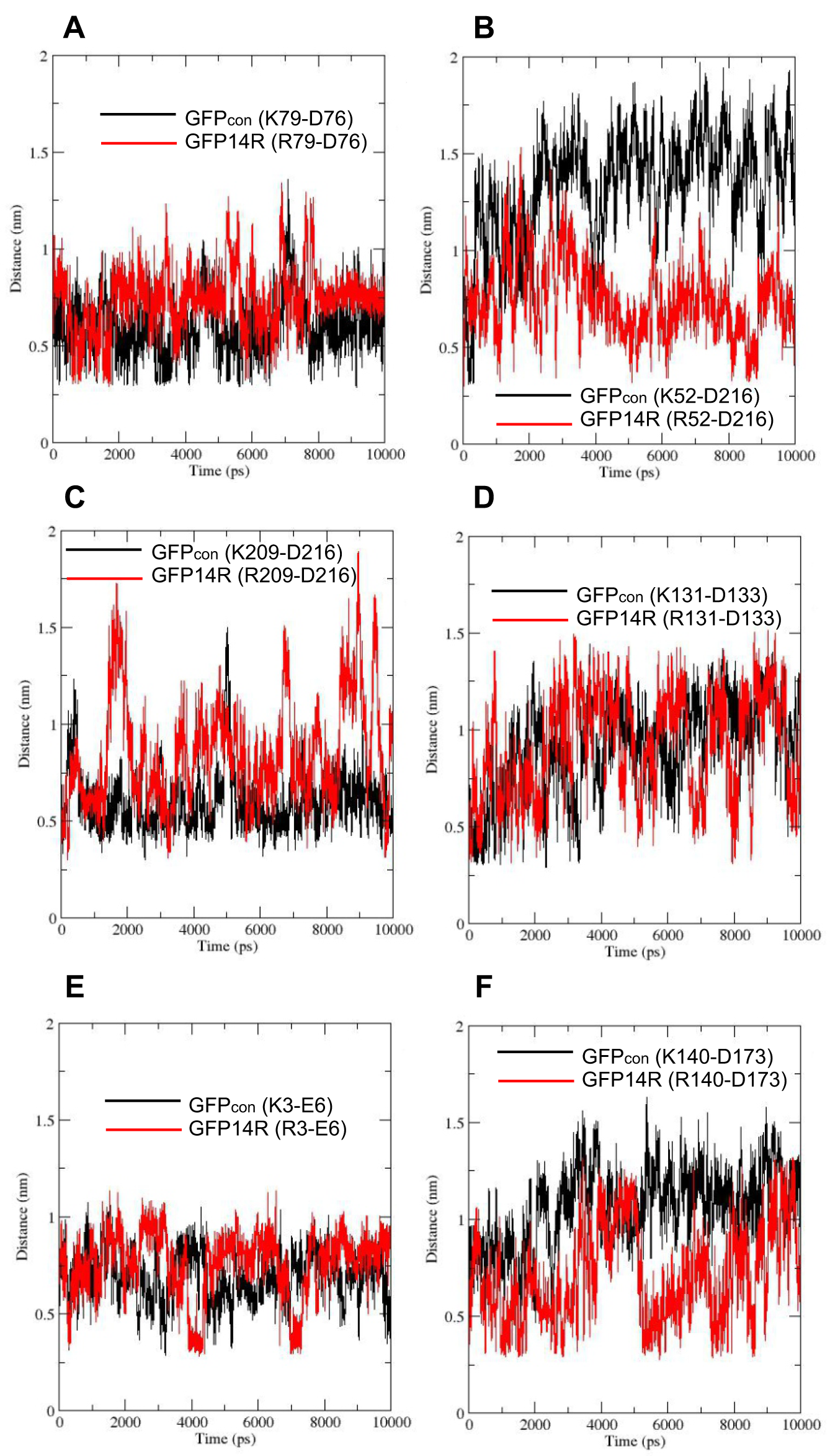

Supplement: Figure S5 — The rmsd (root mean square deviation) distance between the ionic pair atoms over the 10 nanoseconds simulation. (nm – nanometer, ps – picoseconds). (TIF) [file pone.0040410.s005.tif]

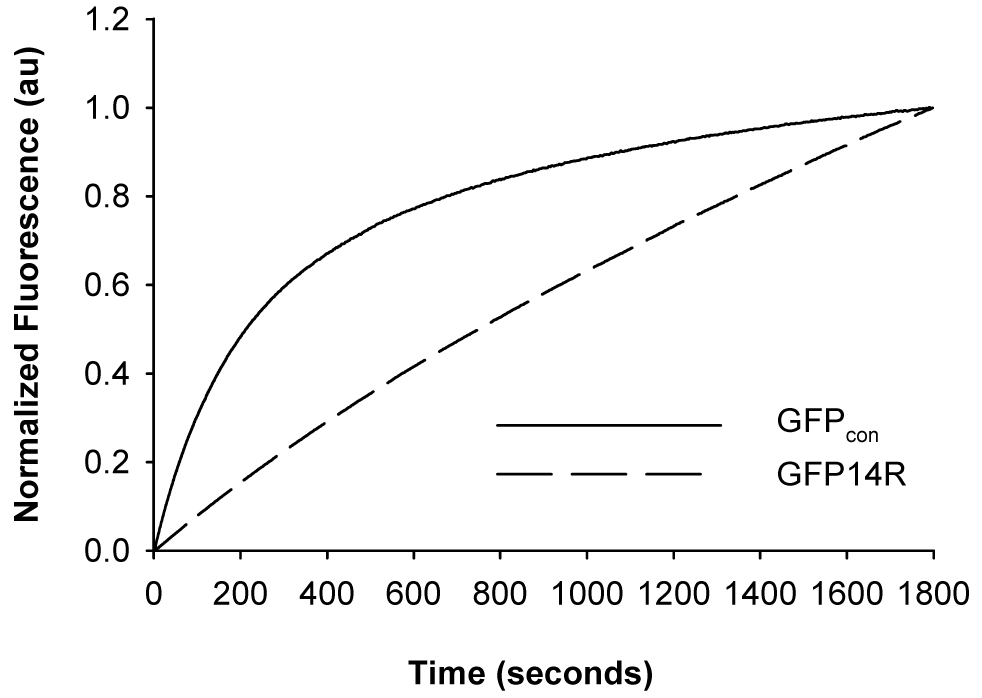

Supplement: Figure S6 — Denaturation and refolding of the GFP variants. The folding efficiency of the GFP variants were measured by denaturing in 8 M urea at 95°C and followed by renaturation by dilution at room temperature. Normalized fluorescence in arbitrary units (au) was plotted against time. (TIF) [file pone.0040410.s006.tif]

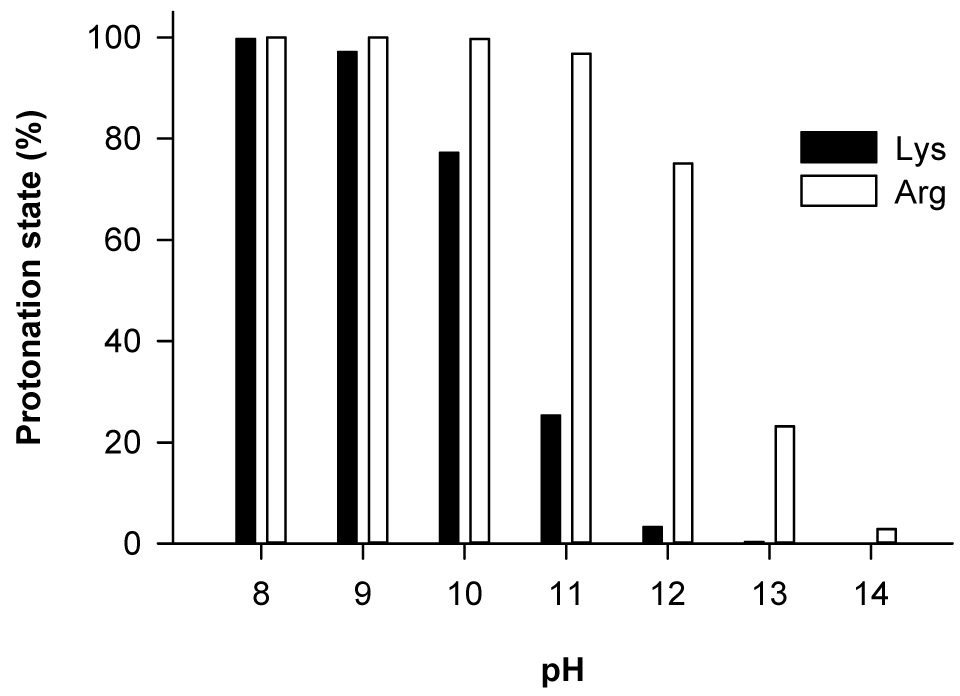

Supplement: Figure S7 — Protonation states were estimated theoretically using the equation log([AH]/[A−]) = pKa-pH. The side chain pKa values of lysine (pKa 10.53) and arginine (pKa 12.48) were used to estimate the ratio of [AH] over [A-] for each pH and converted to percentage. (TIF) [file pone.0040410.s007.tif]

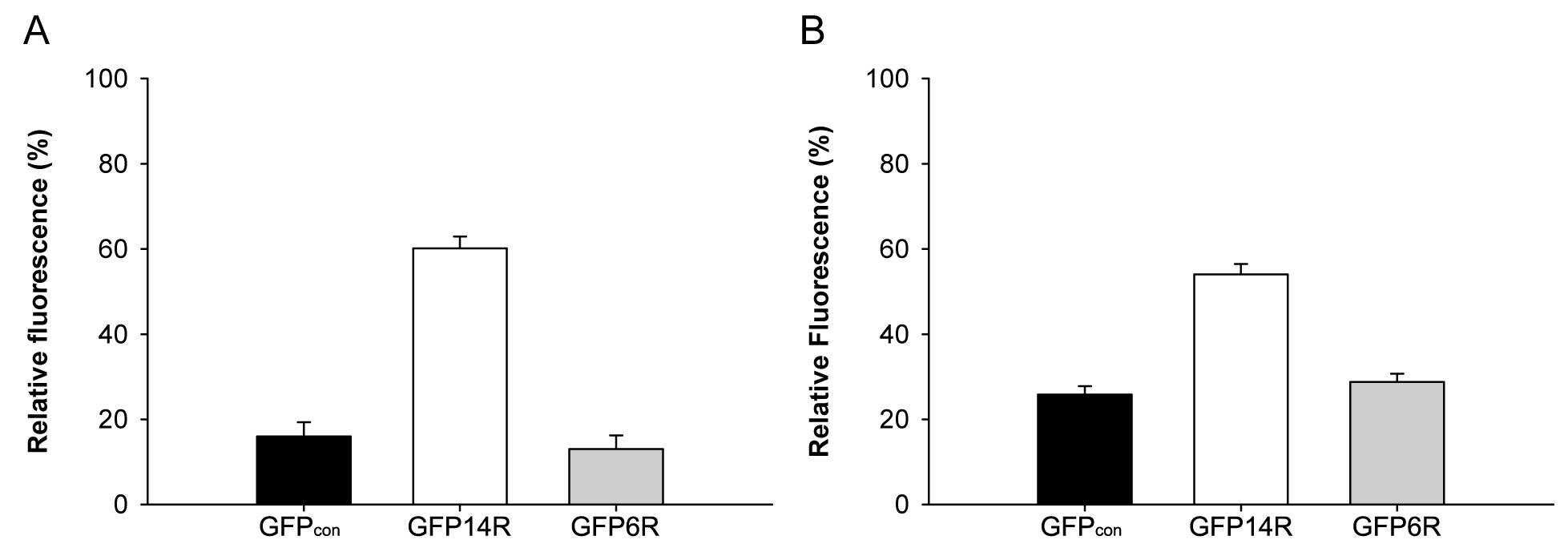

Supplement: Figure S8 — A) Stability of the GFP variants in presence of 1% SDS at 50°C for 30 minutes. The fluorescence at time zero in 1% SDS was taken into 100%. B) Stability of the GFP variants in presence of 50 mM KCl buffer pH 13.0 at 60°C for 30 minutes. The fluorescence at time zero at pH 13.0 was taken into 100%. (Error bar – Standard deviation of the three independent experiments). (TIF) [file pone.0040410.s008.tif]
